# Multiple hybridization events, polyploidy and low postmating isolation entangle the evolution of neotropical species of Epidendrum (Orchidaceae)

**DOI:** 10.1186/1471-2148-14-20

**Published:** 2014-02-04

**Authors:** Isabel Marques, David Draper, Lorena Riofrío, Carlos Naranjo

**Affiliations:** 1 Department of Agriculture (Botany), High Polytechnic School of Huesca, University of Zaragoza, C/ Carretera de Cuarte Km 1, Huesca E22071, Spain; 2 Departamento de Ciencias Naturales, Universidad Técnica Particular de Loja, San Cayetano Alto s/n, Loja 1101608, Ecuador; 3 Current address: UBC Botanical Garden & Centre for Plant Research, and Department of Botany, University of British Columbia, 3529-6270 University Blvd, Vancouver BC V6T 1Z4, Canada

**Keywords:** AFLPs, Conservation, Genome size, Orchids, Reproduction, Reticulate evolution

## Abstract

**Background:**

Hybridization and polyploidy are central processes in evolution and speciation. These mechanisms often lead to complex patterns of genetic variation and the creation of novel genotypes, which may establish if they become isolated from gene flow. However, in the absence of reproductive isolation, species boundaries might easily be disrupted. Here, we used a combination of AFLPs, chloroplast DNA markers and flow cytometry to investigate the evolutionary outcomes of hybridization between two endemic Ecuadorian species of *Epidendrum* (*E. madsenii* and *E. rhopalostele*) in three hybrid zones. Postmating isolation was also quantified to determine the role of this barrier in restraining gene flow between hybrids and the parental species. In addition, future ecological niche models were constructed to predict the outcomes of hybridization between these species.

**Results:**

Our results confirmed the presence of hybrids in all hybrid zones, but revealed that a third parental species (*E. falcisepalum*) has contributed to one of the hybrid zones studied. Backcross genotypes were frequent in all hybrid zones, which was in accordance with the absence of strong reproductive barriers. The process of hybridization was highly asymmetric and followed in some cases by polyploidy. The projection of future niche models predicted a severe reduction in the area suitable for the occurrence of these species, although favorable conditions will still occur for the existence of the current hybrid zones.

**Conclusions:**

The recurrent process of hybridization has compromised the genetic integrity of the parental species. Most individuals of the parental species can no longer be considered as pure-bred individuals because most were classified as backcrossed hybrids. Novel genetic lineages occur in all hybrid zones implying that hybrids are fertile and can compete with the parental species. These results, together with the prediction of suitable conditions for the future occurrence of these hybrid zones, highlight the importance of conserving these geographic areas as sources of novel taxonomic entities.

## Background

Natural hybridization has been reported to occur in a variety of organisms [1], and is often associated with areas where previously isolated lineages come into contact and mate, producing offspring of mixed ancestry [2]. If hybrids are formed easily, a hybrid zone may develop, which may persist over time [3] or lead to the fusion of parental species into a single interbreeding species [4,5].

Introgression with one or both parental species can ease the progress of genetic swamping [6,7], which coupled with hybrid heterosis [8,9] can further enhance the process of homogenization or displacement of parental species. In some cases, introgressed genotypes colonize new habitats or become genetically stabilized, providing a pathway to the evolution of new lineages [10]. Conversely, strong isolation or strong selection against hybrids can facilitate assortative mating and reinforce reproductive barriers between parental hybridizing species [11,12].

The frequency and success of interspecific matings therefore influence the dynamics and evolutionary outcomes of hybridization. In some contact zones, intermediate hybrid genotypes predominate (‘unimodal hybrid zones’; [13,14]), whereas in others, hybrids are rare and populations consist largely of individuals genetically similar to one or other parental genotype (‘bimodal hybrid zones’; [15]). Nonetheless, studies in several different organisms demonstrate that genotype frequencies may vary considerably within the same hybrid zone and create a continuum from unimodal to bimodal genotype frequencies [16,17]. Finally, species might geographically overlap but never hybridize, mostly because of strong prezygotic isolation [18].

Understanding the strength of reproductive barriers might provide important clues concerning the evolution of hybrid zones [19,20]. Prezygotic barriers are usually considered to be most important in the process of speciation [21-24]. Asymmetries in the flowering period of co-occurring species or specificity of pollinators are among the most important prezygotic barriers [25] and, indeed, might shape the structure of hybrid zones. For instance, bimodal hybrid zones are usually characterized by having strong prezygotic barriers, in contrast to their weakness in unimodal hybrid zones [17]. However, postzygotic barriers might also influence the outcome of hybridization, especially in cases where prezygotic barriers are incomplete and allow frequent homogenizing gene flow [26,27].

Polyploid formation, which was formerly expected to promote immediate reproductive isolation between the new incipient species and its progenitors [22], is nowadays considered to be more complex than originally thought [28]. For instance, autotetraploids of *Chamerion angustifolium* are not instantly isolated from their diploid progenitors, although isolation might arise in time [29,30]. Furthermore, asymmetric gene flow from the diploid parent *Capsella rubella* to its allotetraploid progeny *C. bursa-pastoris* has been demonstrated and contributes significantly to high genetic variation in the novel polyploid [31]. Because a single origin of a polyploid represents an extreme bottleneck in this complex pattern of speciation, multiple scenarios might also be invoked to understand polymorphism across ploidy levels such as for instantaneous, recurrent polyploidization or reproductive isolation of polyploids [31].

Orchid species (Orchidaceae) are particularly prone to hybridization most likely because of the high number of sympatric species, in combination with a general lack of complete reproductive barriers [10]. Many studies, especially those devoted to Mediterranean orchids, support this notion, and have provided many examples of how hybridization between orchids is influenced by several prezygotic [32,33] and postzygotic barriers [34]. In addition, vegetative spread and polyploidy may contribute to stabilization of many of these hybrids [35]. Outside the Mediterranean region, one of the best-known examples where hybridization is thought to have a strong influence in diversification is the genus *Epidendrum*. It is the largest orchid genus in the Neotropics and comprises about 1500 species distributed from the United States to Argentina [36]. Plants of intermediate morphology occur in many populations and the existence of many hybrid zones have been reported based on morphological data and geographical overlap between different species of *Epidendrum*[36-38]. Understanding the process of hybridization in *Epidendrum* based on morphological characters is a challenging task owing to the high variability of species, which often hinders reliable identification of species in the field [38]. However, despite the general belief that hybridization is frequent, only one molecular study has reported the consequences of hybridization in *Epidendrum*[39].

In this study, we sought to understand the dynamics of gene flow between *Epidendrum madsenii* Hágsater and Dodson and *E. rhopalostele* Hágsater and Dodson, two epiphytic deceptive orchids that co-exist in three hybrid zones in southern Ecuador [36]. *Epidendrum madsenii* and *E. rhopalostele* belong to two different taxonomic groups and are well differentiated by their morphological traits [36]. The occurrence of hybridization between these species has been postulated on account of the existence of individuals with intermediate morphological traits in some areas of sympatry (Riofrío *et al., unpub. data*). Most species of *Epidendrum* have low pollinator specificity, which together with a high reproductive compatibility even between unrelated species suggests that hybridization may be common [39]. The main question addressed in this study was whether morphologically intermediate individuals were indeed hybrids between *E. madsenii* and *E. rhopalostele*, or simply fell within the wide range of morphological variation shown by these species. Given that other congeneric species are also present in the same area and these species are pollinated by generalist insects, the possibility that different parental species might give rise to morphologically similar hybrids must also be considered [40].

To answer these questions, we investigated the genetic structure of the three known hybrid zones and the nature of the morphologically intermediate specimens using an approach that combined AFLPs, cpDNA markers, genome size and controlled pollinations in conjunction with climate-based predictive models. The results allowed us to (1) characterize the genomic composition of hybrid zones and their hybrids (e.g., F_1_ hybrids and backcrosses); (2) determine the presence of asymmetrical hybridization patterns and the dynamics of genome size in different hybrid zones; (3) investigate the strength of reproductive barriers in different hybrid zones; and (4) based on the molecular data and niche models, predict the long-term fate of the hybrid zones between these deceptive orchid species.

## Results

### Plastid DNA diversity and haplotype network

**Figure 1:**
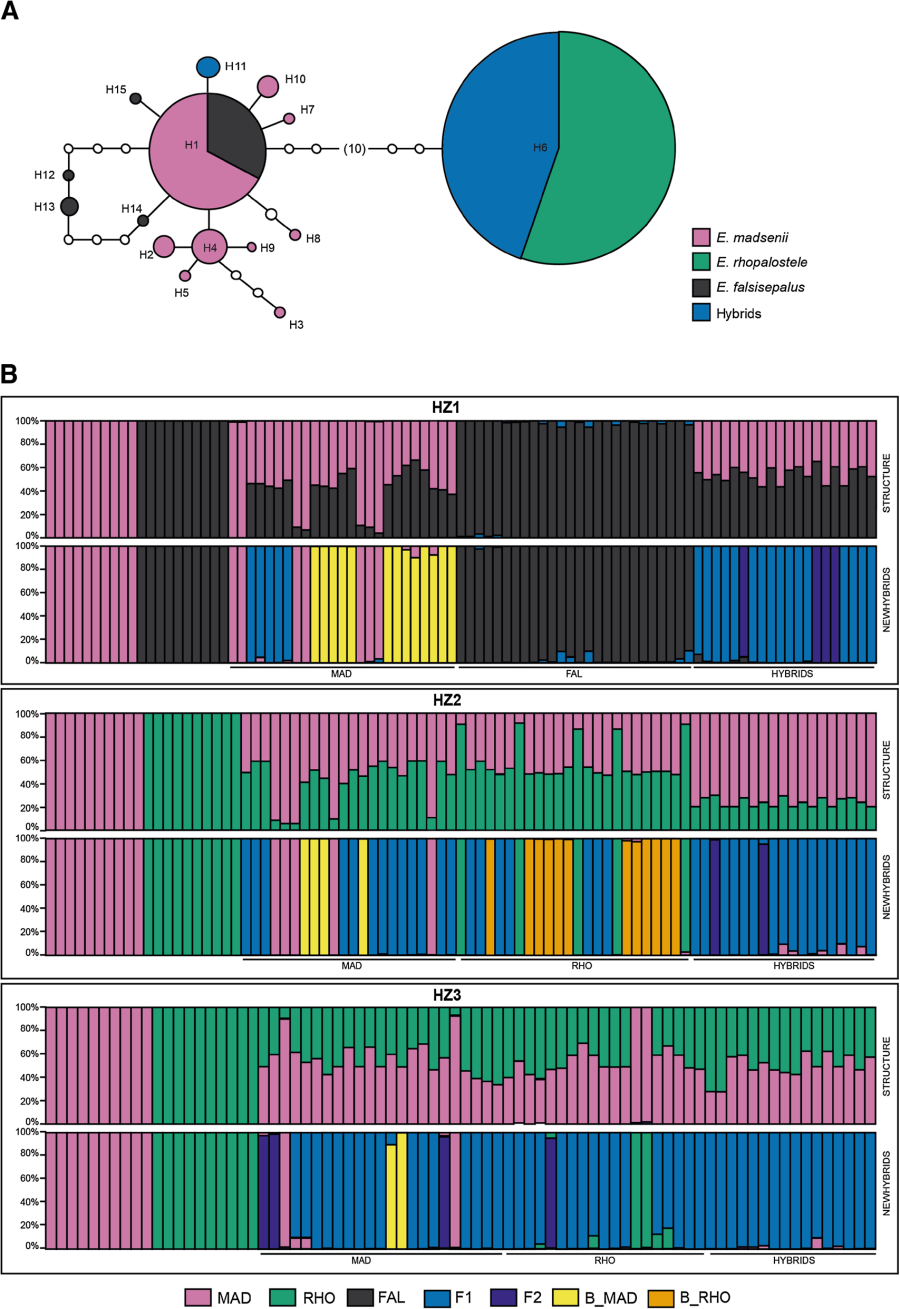
**Genetic variation in *E. madsenii*, *E. rhopalostele*, *E. falcisepalum *and their hybrids across three hybrid zones. (A)** Statistical parsimony network of plastid haplotypes based on sequences from six chloroplast DNA regions (*trnL-trnF*, *rps16*, *rpoC1*, *psbK-psbI*, *matk* and *rbcl*). A circle’s size is proportional to the haplotype frequency. Small empty circles represent single mutational steps. **(B)** Posterior probabilities (*q*) for the three hybrid zones of *Epidendrum madsenii* ‘MAD’ and *E. falcisepalum* ‘FAL’ (HZ1) and *E. madsenii* ‘MAD’ *E. rhopalostele* ‘RHO’ (HZ2 and HZ3) analyzed with STRUCTURE and NEWHYBRIDS. Individuals identified in the field, based on morphological characters, are delimited by dashed lines. Each vertical bar represents an individual. The proportion of color in each bar represents an individual’s assignment probability, according to different categories (pure parental species, F_1_ and F_2_ hybrids, and the respective backcrosses).

Statistical parsimony analysis yielded a single network containing two major groups with 15 cpDNA haplotypes: one comprising all sequences for *E. madsenii* and *E. falcisepalum* F. Lehm. & Kraenzl., and the other grouping *E. rhopalostele* and most putative hybrids in a single haplotype (Figure 1A, Additional file 1: Table S1). The highest haplotype diversity was observed in *E. madsenii*, which had nine haplotypes, whereas the lowest diversity was shown by *E. rhopalostele*, which only had one haplotype (H6). The predominant haplotype in *E. madsenii* (H1) was also shared with *E. falcisepalum*. Three additional haplotypes were observed in *E. falcisepalum*. The majority of hybrids shared the haplotype H6 with *E. rhopalostele* (83%), whereas the remaining hybrids contained a unique haplotype (H11; Figure 1A).

GenBank accession numbers and sequence statistics are given in Additional file 2: Table S2 and Additional file 3: Table S3, respectively. Hybrids usually showed higher nucleotide diversity than their progenitor species (Additional file 3: Table S3).

### Genetic composition of hybrid zones

Bayesian assignment results obtained with STRUCTURE confirmed that the allopatric parental populations were exclusively composed of pure genotypes because the assignment results were very high with a threshold *q*-value ≥ 0.90 (Figure 1B). However, in all hybrid zones, more than half of individuals morphologically classified in the field as parental species showed intermediate *q*-values (0.10 ≤ *q* ≤ 0.90) with STRUCTURE (Figure 1B).

Because genome size (GS) values suggested the involvement of *E. falcisepalum* and *E. madsenii* in the origin of hybrids in HZ1 (see below), Bayesian assignment tests of this hybrid zone were run with these species (instead of *E. rhopalostele*) and hybrids indeed showed intermediate *q-*values in this population (Figure 1B). Surprisingly, *E. falcisepalum* was indicated to be descended from backcrosses with *E. madsenii* (Additional file 4: Figure S1), which is consistent with the phylogenetic proximity of the two species.

The individuals revealed by STRUCTURE to be admixed were assigned to one of the different hybrid classes with NEWHYBRIDS, although the genetic composition varied across hybrid zones. Individuals morphologically classified as *E. madsenii* were predominantly assigned as backcrossed hybrids in HZ1 and F_1_ hybrids in HZ2 and HZ3. In contrast, individuals morphologically classified as *E. rhopalostele* were assigned as backcrossed hybrids in HZ2 and as F_1_ hybrids in HZ3, whereas individuals morphologically classified as *E. falcisepalum* was assigned as a pure parental species in HZ1 (Figure 1B).

Hybrids were predominantly assigned as F_1_ hybrids, although F_2_ individuals were also present in all populations (Figure 1B).

### Genome size

**Figure 2:**
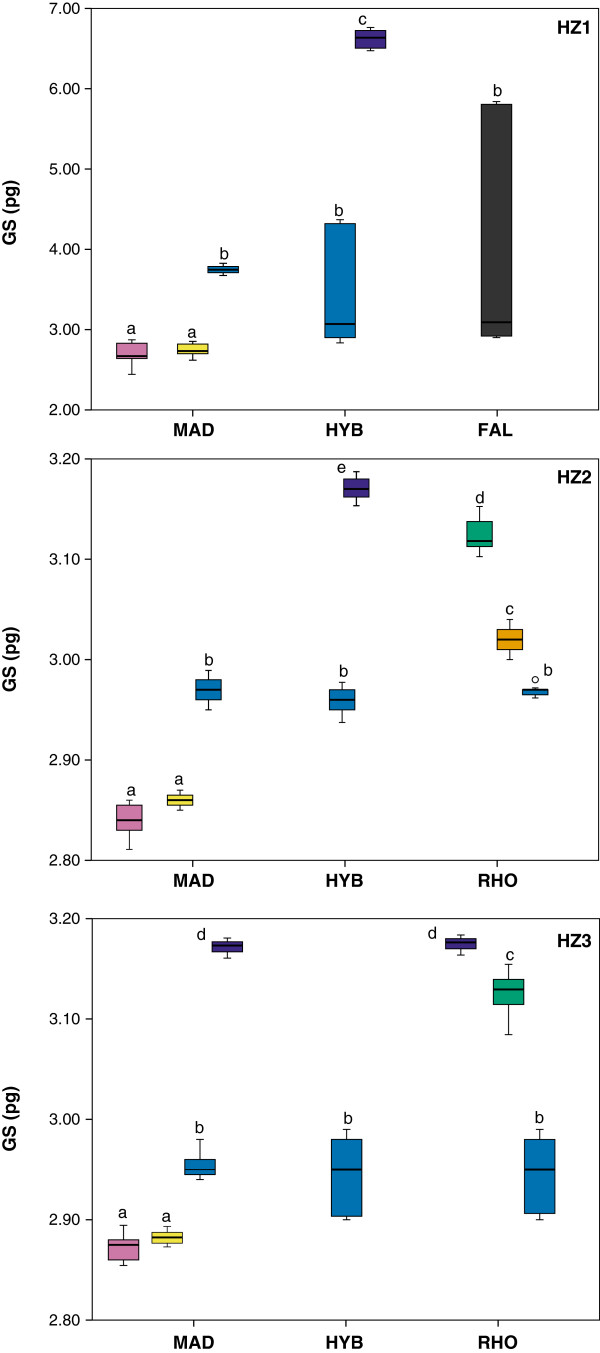
**Genome size obtained with PI for three hybridizing species of *Epidendrum*: *E. madsenii *‘MAD’, *E. rhopalostele *‘RHO’ and *E. falcisepalum *‘FAL’ in three hybrid zones (a-c) and in allopatric populations (d-f) according to the genetic groups detected by NEWHYBRIDS (colors as in Figure **1**B).** Values are expressed in picograms.

GS values were significantly different between the three pure parental species and the mean values were 2.69 pg in *E. madsenii*, 3.13 pg in *E. rhopalostele* and 4.08 pg in *E. falcisepalum* (*F*_3,250_ = 19.199, *P* < 0.001). Analyzing the individuals according to the genetic groups assigned by NEWHYBRIDS allowed us to determine that the GS of F_1_ hybrids conformed to expectations (GS parent1 + GSparent2/2) as they usually showed values intermediate to those of the parental species in all hybrid zones (Figure 2). Backcrossed individuals were usually grouped with the respective backcrossed parental species, and F_2_ hybrids had GS values higher than those of the remaining genetic groups (Figure 2).

Some individuals of *E. falcisepalum* had a GS of 2.91 pg, whereas other individuals showed a value of 5.84 pg, which suggested the occurrence of polyploidy in this species (Figure 2). For the parental species, no significant differences were observed between the GS values of allopatric individuals and those assigned by NEWHYBRIDS as pure in the three hybrid zones (*t* = 0.117, df = 98, *P* = −0.026 for *E. madsenii*; *t* = −0.026, df = 64, *P* = 0.979 for *E. rhopalostele*; *t* = 0.296, df = 34, *P* = 0.769 for *E. falcisepalum*).

### Postmating prezygotic isolation: formation of fruit

**Figure 3:**
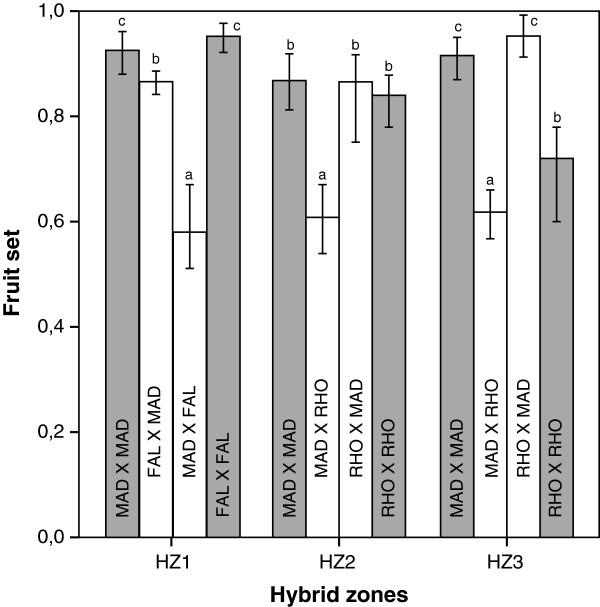
**Mean fruit set in experimental crosses within (grey bars) and between (white bars) the studied species of *Epidendru*m.** Values represent the mean ± SD (*N* = 100 plants⁄cross). The first letters indicates the identity of the maternal species: FAL = *E. falcisepalum*; MAD = *E. madsenii*; RHO = *E. rhopalostele*. Crosses with the same letter do not differ significantly (*P* > 0.05).

Experimental pollinations between the pure parental individuals assigned by NEWHYBRIDS yielded different results among the three hybrid zones. In HZ1, interspecific fruit set was lower than intraspecific fruit set, especially when *E. madsenii* was the ovule donor (Figure 3, Table 1). In HZ2 and HZ3, no differences were observed between intra- and interspecific crosses when *E. rhopalostele* was the ovule donor, whereas interspecific fruit set was slightly lower than intraspecific fruit set when *E. madsenii* was the ovule donor (Figure 3, Table 1). No differences were observed between populations in the interaction of population × treatment (Table 1). The strength of postmating prezygotic isolation (RIpostmating/prezygotic) was therefore very low, but variable among species (Table 2).

**Table 1:** **Effects of pollination treatment and population on fruit set and seed viability of *Epidendrum***

**Frui set**					
	**SS**	**df**	**MS**	***F***	***P***
Population	0.1671	2	0.083	0.028	0.383
Treatment	11.021	3	3.673	1.252	4.604 E-13
Population × treatment	0.881	6	0.146	0.050	0.872
Error	23.457	8	2.932		
**Seed viability**					
	**SS**	**df**	**MS**	***F***	***P***
Population	0.261	2	0.130	0.330	0.894
Treatment	0.335	3	0.111	0.282	0.970
Population × treatment	0.672	6	0.112	0.280	0.860
Error	3.561	9	0.395		

**Table 2:** **Strength of postmating prezygotic isolation (RIpostmating/prezygotic) in the different hybrid zones (HZ) studied for the three species of *Epidendrum*: *E. falcisepalum *(FAL), *E. madsenii *(MAD) and *E. rhopalostele *(RHO)**

	**FAL**	**MAD**	**RHO**
HZ1	0.07	0.40	-
HZ2	-	0.30	0
HZ3	-	0.27	0

### Postmating postzygotic isolation: development of viable seeds

Among all species, the percentage of viable seeds was 78.91% ± 25.43 (mean ± SD). No significant difference was observed between intra- and interspecific crosses or between populations (Table 1). Consequently, the strength of this postmating postzygotic barrier (RIpostzygotic) was null in all populations.

### Niche models

**Figure 4:**
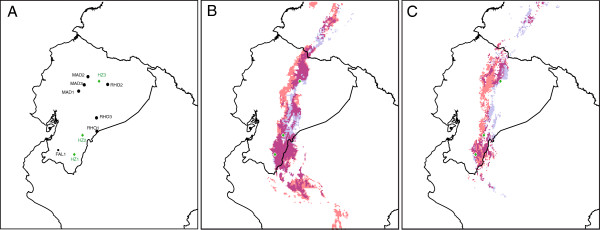
**Localities sampled in this study (A); Green dots indicate the 3 hybrid zones studied (HZ) while black dots indicate the allopatric populations of *E. falcisepalum *(FAL)*, E. rhopalostele *(RHO) and *E. madsenii *(MAD).** Predictive ecological model of current distribution **(B)** and the future scenario **(C)** based on the Maxent algorithm of *Epidendrum madsenii* (blue) and *E. rhopalostele* (red). The different maps assess similarity of niche models between the two species using the intersection of both MPA species. Predominance of one color indicates niche differentiation whereas a dark color indicates overlapping of niche models.

The present bioclimatic niche climate was consistent with the currently known distribution of *E. madsenii* and *E. rhopalostele* (Figure 4 and Additional file 5: Figure S2). The area under the curve (AUC) score for these models was very high (0.88 for *E. madsenii* and 0.91 for *E. rhopalostele*). The projection of the future bioclimatically suitable area showed a general shift of the current predicted range because most southern favorable areas will disappear (Figure 4). In addition, a severe reduction in the area predicted to be suitable for the occurrence of *E. madsenii* and *E. rhopalostele* was shown (Figure 4).

Both species will lose more than half of their current distribution area between the present day and 2080 (from present 56,238 km^2^ to future 29,131 km^2^ in *E. madsenii* and from 71,787 km^2^ to 37,439 km^2^ in *E. rhopalostele*). Consequently, the future scenario showed a decrease of 43.2% in the minimal predicted area (MPA) intersection of *E. madsenii* and *E. rhopalostele* (from 43,260 km^2^ to 16,827 km^2^; Figure 4), which suggested a decrease in the geographical area potentially suitable for hybridization. Nevertheless, the future scenario reveals the presence of suitable conditions for the occurrence of the three hybrid zones studied here (Figure 4).

## Discussion

### A complex scenario of reticulate evolution in Neotropical orchids

This study has shown conclusively that a recurrent series of interspecific hybridization events and backcrosses among closely related taxa has lead to the generation of genetic novelty and to a complex pattern of ongoing reticulate evolution within *Epidendrum*. Our molecular, reproductive and genome size data indicate that three different species have given rise to two morphologically similar hybrids in different areas of Ecuador. In two of the hybrid zones studied (HZ2 and HZ3), hybrids were derived from *E. madsenii* and *E. rhopalostele*, whereas in a third area (HZ1), hybrids were instead derived from *E. madsenii* and *E. falcisepalum*, of which the latter species is also of hybrid origin. The bayesian tests assigned *E. falcisepalum* as a backcrossed hybrid with *E. madsenii* and genome size values are consistent with the presence of chromosome duplication as a result of polyploidy. Thus, not only different species were involved in the process of hybridization but in some cases it was also followed by polyploidy. Although our preliminary chromosome counts indicate that the three species are diploid (2*n* = 28 chromosomes; results not shown), we were unable to count the chromosomes of all individuals studied, and the differences in genome size observed could represent different chromosome numbers.

Our data indicate that *E. rhopalostele* was in most cases the maternal progenitor of the hybrids, except in the population HZ1 where *E. madsenii* seemed to have acted as the ovule donor. The fact that most cpDNA sequences were shared with these species is consistent with this hypothesis. The predominance of these two species as the maternal progenitor (in HZ1 for *E. madsenii*, and in HZ2 and HZ3 for *E. rhopalostele*) is also consistent with the results of controlled pollinations because fruit set was significantly higher when these species acted as the ovule donor.

Hybridization is therefore highly directional and leads to the asymmetric formation of hybrids in nature, as reported in other studies [41]. Asymmetric hybrid formation is not unusual in nature [42], and can be caused by complex genotype–environment interactions [43] or complete cytoplasmic incompatibility [44]. Differences in the flowering period of co-occurring species might also explain the presence of a unidirectional gene flow in hybridizing populations [45]. Although preliminary, an ongoing study reveals differences in the flowering patterns of these populations (Vega, pers. comm.).

### Outcomes of hybridization in *Epidendrum*

Although hybridization has been long postulated to be an important evolutionary mechanism in *Epidendrum*, genetic confirmation of the presence of hybrids in this genus has been reported only in the last 3 years and, although results are limited to two examples, hybridization is believed to be a frequent phenomenon [46,47]. For example, the use of nuclear and plastid microsatellites revealed high potential for interspecific gene flow even between species with two different ploidy levels, i.e. *E. fulgens* (2*n* = 2*x* = 24) and *E. puniceoluteum* (2*n* = 4*x* = 52) [39]. Although results suggested the presence of male sterility in F_1_ hybrids and backcrosses, they also revealed wide ecological amplitude of the hybrids [39], which suggested exogenous selection is of low importance in this study case. Furthermore, despite the fact that hybrids were triploids (2*n* = 3*x* = 38 and 40), they were able to backcross and produce viable hybrid lineages [48].

The prevalence of hybridization was also reported in a study comprising 25 sympatric populations of *E. calanthum*, *E. cochlidium* and *E. schistochilum*[49]. These three diploid species (2*n* = 28) frequently hybridize when they co-occur, although outcomes depend on the species involved. F_1_ hybrids were frequent in the sympatric populations of *E. cochlidium* and *E. schistochilum*, whereas in the remaining populations backcrosses with *E. calanthum* were predominant when this species co-occurred with *E. cochlidium*, but when it co-occurred with *E. schistochilum* backcrosses with the latter species prevailed [49].

In contrast to the previous studies, the presence of hybrid genotypes was predominant in all sympatric populations and most parental species were no longer considered as pure species [49]. The same results were observed in the current study, especially in the hybrid zones where *E. madsenii* and *E. rhopalostele* co-existed. A high number of viable seeds were produced in our experimental crosses and populations were not exclusively composed of F_1_ hybrids, which would be the case if the hybrids were sterile. The existence of one specific haplotype in some hybrids (H11; Figure 1) is also consistent with the fact that populations are not composed only of early-generation hybrid genotypes, which contrasts with most studies of hybrid zones between deceptive orchids [39,50,51]. Rather, populations were also composed of F_2_ hybrids and most likely of backcrossed hybrids.

Given that the two hybrids studied here are fertile, they can easily form a bridge for gene flow between the parental species [52]. The studied species are deceptive orchids and pollinators usually do not strongly discriminate species [39], thus they contribute to introgressive hybridization through creation of a hybrid swarm and the possible merging of species [53]. Probably many other examples of hybrid zones among deceptive orchids exist, but molecular studies documenting the genetic structure of these hybrid zones are scarce, especially in the case of Neotropical orchids (but see [39]).

### Evolution of reproductive barriers in *Epidendrum*

The high frequency of hybridization in all populations studied suggests the general absence of strong interspecific reproductive barriers. Although not studied here, premating barriers are apparently weak in our sympatric populations owing to the high frequency of hybrid genotypes observed. In accordance with this conclusion, several studies have revealed that *Epidendrum* is usually pollinated by a wide number of species, which do not show any pattern of fidelity to a given flower species (reviewed in [47]). Hybridization and late-generation hybrids are frequent in all *Epidendrum* species surveyed, even between species with different ploidy levels, and although some degree of sterility is observed, there is always a proportion of fertile hybrids with high fitness in a broad amplitude of habitats [39]. However, some caution is needed when considering the generally low importance of reproductive barriers in *Epidendrum* because our knowledge of the frequency and outcomes of hybridization is still limited, even in the species studied. For instance, we only found three hybrid zones even though the species studied here could apparently co-exist in more sites (Figure 4). Furthermore, no studies have evaluated the role of exogenous selection in the establishment of hybrid genotypes or its association with the mycorrhizal community.

The occurrence of polyploidy, which has been previously suggested to occur in this genus [38], was confirmed in our study. Besides hybridization, polyploidy offers a good explanation for the high number of *Epidendrum* species described. Allopolyploid speciation (duplication of chromosomes in hybrids) may occur in these orchids by several processes, such as somatic chromosome doubling followed by selfing to produce a tetraploid, through fusion of two unreduced gametes, or by backcrossing of triploids with haploid gametes to form tetraploid progeny [10].

### Can we deal with taxonomic homogenization and blurring of species boundaries?

The widespread hybridization revealed in this species complex raises several taxonomic and conservational concerns, because it may compromise the genetic integrity of the parental species to the point of causing local extinctions. This is exacerbated by the finding that future niche models predict a severe decrease in the area favorable for the occurrence of the parental species.

With the exception of the polyploid *E. falcisepalum*, which seems to escape the homogenizing effects of gene flow, the remaining parental species can no longer be seen as pure-breeding species because they consist of predominantly backcrossed individuals (*E. madsenii* in HZ1 and *E. rhopalostele* in HZ2) or F_1_ hybrids (*E. madsenii* in HZ2 and HZ3, and *E. rhopalostele* in HZ3). The virtual absence of pure-bred individuals in these hybrid zones implies that genetic swamping has eliminated the parental species and led to the loss of species identity [53]. It also highlights the fact that, besides morphological characters, other taxonomic tools such as molecular markers should be used in order to understand species boundaries in orchids.

Previous studies have demonstrated the existence of introgression in plants [54-57], which in some cases has led to the homogenization of species characteristics and the extinction of parental species in very few generations. For instance, *Helianthus bolanderi* suffered a rapid reduction in its population size owing to genetic swamping by the common invasive *H. annuus*[6]. Asymmetric introgression also occurs between *Iris fulva* and *I. brevicaulis* where 72% of the *I. brevicaulis*-like plants have alleles introgressed from *I. fulva*[58].

Understanding if such consequences are derived from natural or anthropogenically mediated hybridization is important for species management and conservation. Allendorf *et al.*[59] categorized hybridization into six types based upon the extent of hybridization and the natural or anthropogenic status of the hybridization. However, in the present study and most other cases it is difficult to know whether hybridization was triggered by natural causes or not. In some cases, it can even be argued that human interventions simply amplify the potential for hybridization (e.g., through long-distance dispersal) or create environments that are more suitable for the new hybrid lineages [60].

In *Epidendrum*, hybridization appears to be mainly the result of natural causes and is frequently caused by the lack of pollinator specificity and the absence of strong reproductive isolation barriers [39]. Therefore, in biological groups where hybridization is so frequent and creates successful interbreeding lineages, two questions arise concerning what to do with the species involved. Can we even consider them as “species”? Does it make sense to restore the historical genetic structure if threatened species are involved or should we instead target our efforts to the new genetic novelties?

Based on future projection of their ecological niche, and given that conditions suitable for these hybrid zones will exist, the hybrid zones can therefore be seen as a source of raw material for natural adaptive change [61,62]. In this light, the definition of species should better focus on traits that lead to adaptation and conservation efforts should be targeted towards evolutionary processes that generate taxonomic biodiversity instead of preserving the taxonomic entities beyond these processes [63]. Although this strategy cannot be applied to all organisms, it certainly provides a good framework for determining evolutionarily important units [63,64] that are worthy of protection and management in species with complex reticulate scenarios, such as those in the present investigation.

## Conclusions

Although hybridization is usually invoked as a driving force for the high levels of morphological variation in *Epidendrum*, the existence of hybrid zones and the levels of genomic admixture remain unknown in most cases. Here, we demonstrate that hybridization can be frequent whenever two species co-occur, leading to a complex scenario of reticulate evolution and the blurring of species boundaries. Given that a high number of viable and fertile hybrid seeds were produced, hybridization generated genetic novelties in all hybrid zones. The high frequency of hybrids and the fact that the parental species were mostly assigned as backcrossed hybrids suggests low efficiency of reproductive barriers, at least in these populations. This conclusion is consistent with the general lack of pre- and postmating barriers reported in this genus and, therefore, the large proportion of backcrosses observed might also be a feature of other Neotropical hybrid zones of *Epidendrum*. The high frequency of hidden hybrid genotypes described in this study suggests that more genetic studies are needed to understand the evolution of the genus *Epidendrum*.

## Methods

### Plant sampling

*Epidendrum madsenii* and *E. rhopalostele* are two epiphytic orchids patchily distributed in the Andean tropical rainforest. *Epidendrum madsenii* is endemic to Ecuador and occurs in several disjunct localities on the eastern slope of the Andes, and *E. rhopalostele* is near-endemic, as it occurs along the Andean Cordillera from Central Ecuador to its border with Perú [36]. Flowering is reported to occur from November to May in *E. madsenii* and from May to September in *E. rhopalostele*[36]. The sampling was designed to cover the whole geographic distribution of these species of *Epidendrum* (Additional file 6: Table S4; Figure 4). All reported hybrid zones were sampled (HZ1, HZ2, and HZ3: Additional file 5: Figure S2) and, despite intensive fieldwork, no additional hybrid zones were found. In addition, *E. falcisepalum* found in one of the populations (HZ1) was also sampled because this species could also be considered as a putative progenitor of the hybrids either by flowering phenology or morphological traits. Three allopatric populations of each species (MAD1, MAD2, and MAD3 in the case of *E. madsenii*, and RHO1, RHO2, and RHO3 in the case of *E. rhopalostele*) were also included in the study as reference populations (except *E. falcisepalum* for which only one allopatric population was found: FAL1; Additional file 5: Figure S2). In total, 286 individuals were tagged and sampled: 100 morphologically assigned as *E. madsenii* (70 from the hybrids zones – HZ1:25, HZ2:22, HZ3:23 – and 30 from allopatric populations – MAD1:10, MAD2:10, MAD3:10), 95 morphologically assigned as *E. rhopalostele* (65 from the hybrids zones – HZ1:21, HZ2:24, HZ3:20 – and 30 from allopatric populations – MAD:10, MAD2:10, MAD3:10), 55 putative hybrids (HZ1:20, HZ2:19, HZ3:16) and 36 morphologically assigned as *E. falcisepalum* (26 from the hybrids zones HZ1 and 10 from the allopatric population FAL1; Additional file 6: Table S4). Given that these species can propagate by vegetative reproduction, individuals were collected with a minimum sampling distance of 10 m. Voucher specimens were deposited at the herbarium of the Universidad Técnica Particular de Loja. Field studies were conducted in accordance with local legislation. Samples were preserved as silica-gel dried leaves and stored at −80°C until use for DNA extraction.

### Molecular methods

#### DNA isolation, AFLP procedure and sequencing

For the 286 individuals sampled, DNA was extracted using the DNeasy™ Plant Minikit (Qiagen, Hilden, Germany) following the manufacturer’s instructions and stored at −20°C.

Procedures for AFLP analysis basically followed the protocol developed by [65]. An initial screening of 32 combinations of selective primers was performed using five individuals from different populations. Three combinations were selected because they yielded clear and evenly distributed bands: EcoRI-AC (FAM)/MseI-CTA, EcoRI-AG(FAM)/MseI-CTT and EcoRI-AGG(VIC)/MseI-CTC.

A reproducibility test was performed by re-extracting DNA from two plants per species and per population and repeating the whole AFLP procedure. The error rate was calculated for every primer combination as the ratio of mismatches (scoring of 0 vs 1) over phenotypic comparisons in AFLP profiles and subsequently averaged over the three combinations. Non-reproducible fragments were excluded from the analyses.

PCR amplifications were performed for 127 individuals using an Eppendorf thermocycler in 50 μL reactions containing 10 ng DNA, 1× PCR buffer (Invitrogen, Sao Paulo, Brazil), 2.5 mM MgCl_2_, 0.5 μl each primer (10 μM) and 1 μl Taq (45–130 μg/ml) using 5–10 individuals per population/species. Six chloroplast regions were analyzed: *trnL-trnF* (primers c and f: [66]), *rps16* (primers rps16F and R: [67]); and four primers from the CBOL Plant Working Group [68]: *rpoC1* (primers 2f and 4f), *psbK-psbI* (primers F and R), *matk* (primers 3 F and 1R) and *rbcl* (primers F and R). Primer sequences and PCR conditions were obtained from the literature (see references above). All PCR products were purified using the UltraClean™ PCR Clean-up™ Kit (MoBio Laboratories, Inc., Carlsbad, CA, USA) in accordance with the manufacturer’s protocol. Amplifications were performed using the original amplification primers. Purified PCR products were sequenced in both directions on a 3730 DNA analyzer (Applied Biosystems, Foster City, CA, USA). The sequencing reactions were performed in a total volume of 10 μL containing 30–50 ng DNA, 5 μM each primer, 2 μL ABI PRISM BigDye Terminator v3.1 cycle sequencing ready reaction kit (Applied Biosystems), and 1 μL of 5× Sequencing Buffer (Applied Biosystems).

#### Data analysis

##### AFLP data

Amplified bands were aligned with the internal size standard using the ABI PRISM GeneScan Analysis Software version 3.1 (Applied Biosystems). Subsequently, fragments of each primer combination were scored automatically with Genographer version 1.6.0 (Montana State University, http://hordeum.oscs.montana.edu/genographer/) either as present (1) or absent (0), and manually corrected. Peaks were recorded in a range from 50 to 500 bp. Highly reproducible AFLP patterns were found for all replicates and the average error rate was estimated as 4.2% across all three primer combinations in agreement with previous studies below 5% [69]. Eight unreliable fragments were removed leading to a total of 223 bands. No identical multilocus phenotypes were observed between individuals or populations.

To infer population structure and the genetic composition of hybrid zones, two Bayesian clustering methods were performed using the software STRUCTURE 2.2. [70] and NEWHYBRIDS version 1.1 beta [71]. Each hybrid zone was analyzed separately using allopatric populations of each species as reference samples of pure individuals. STRUCTURE was first used to classify individuals as either pure parental species or hybrids using a threshold of *q* ≥ 0.90 to assign pure individuals and 0.10 ≤ *q* ≤ 0.90 to classify hybrids, where *q* represents the admixture proportion. We assigned two groups (*K* = 2) in this study because we assumed that two species contributed to the genetic pool of the sample. Calculations were performed running 10 simulations under the admixture model. The same genotype data used in the STRUCTURE analysis was analyzed with NEWHYBRIDS to classify the genetic composition of the hybrids in six different classes: F_1_, F_2_, backcross to each parental species, and pure parental species, using a threshold of 0.75. A burn in of 50,000 steps followed by run lengths of 300,000 were used in each program. Similarity coefficients between runs and the average matrix of ancestry membership were calculated using CLUMPP version 1.1 [72] and visualized using DISTRUCT software [73].

##### Sequence data

Sequence alignment was performed manually using BioEdit 7.0.0 Sequence Alignment Editor [74], which was also used to check electropherograms. DnaSP version 3 [75] was used to characterize DNA polymorphism. Within-species diversity was estimated with Nei’s haplotype diversity (Hd) and in terms of weighted sequence divergence with nucleotide diversity (π) [75]. Chloroplast DNA sequences (the four regions concatenated) were analyzed using statistical parsimony as implemented in TCS 1.21 with gaps coded as missing data [76].

### Genome size

Genome size was measured for the 286 individuals that were genotyped. Fresh leaves of *Epidendrum* were co-chopped with a fresh leaf of *Pisum sativum* (internal reference standard with 2C = 8.76 pg) using a sharp razor blade in a Petri dish containing 1 ml WPB (0.5 mM spermine.4HCl, 30 mM sodium citrate.3H_2_O, 20 mM MOPS, 80 mM KCl, 20 mM NaCl, 0.5% (v/v) Triton X-100, pH adjusted to 7.0). The nuclear suspension was recovered and filtered through a 50 μm nylon filter to remove cell fragments and large debris. Nuclei were stained with 50 mg/ml propidium iodide (Fluka, Buchs, Switzerland), and 50 mg/ml RNase (Sigma, St Louis, MO, USA) was added to the nuclear suspension to prevent staining of double-stranded RNA. Five minutes after staining, the relative fluorescence intensity of at least 3,000 nuclei was analyzed in a Partec CyFlow Space flow cytometer (Partec GmbH., Münster, Germany), equipped with a green solid-state laser for PI excitation, using the FloMax software (Partec GmbH). As a quality control, only when CV values of G0/G1 peaks were below 3% were the analyses saved, otherwise sample preparation was repeated. The mean and standard deviation of the mean (SD) of each sample were calculated. The normality of the distribution of genome size (GS) of all samples was assessed using the Kolmogorov–Smirnov test. Differences in GS between hybrid classes were evaluated using analysis of variance (ANOVA). In those cases in which ANOVA revealed significant differences, the Tukey HSD post-hoc test was performed.

### Assessment of reproductive isolation

Controlled pollinations were performed during January 2012 in the three hybrid zones and using the individuals considered “pure” species by STRUCTURE and NEWHYBRIDS (e.g., *q* > 0.90). Pollinations were also performed in one allopatric population per species (MAD1 for *E. madsenii* and RHO for *E. rhopalostele*). Crosses were performed between individuals of the same species (intraspecific crossings) and between individuals of different species (interspecific crossings). Pollinations were performed by removing pollinia with a plastic toothpick and placing them on the stigmas of other individuals from the same or the other species. Interspecific crosses were performed in both reciprocal directions. To prevent contaminatory pollination, plants were bagged with 1-mm-mesh nylon tulle prior to flowering. A total of 50 randomly selected flowers were used in each treatment. For each inflorescence, two flowers per treatment were used to avoid potential negative effects of over-pollination on fruit set and seed viability. Flowers were monitored for fruit set after anthesis and bagged with 1-mm-mesh nylon tulle to avoid the loss or predation of fruits and seeds. Following [39], samples of 300 seeds per fruit were stained with tetrazolium and analyzed to quantify seed viability. The percentage of viable seeds was determined by dividing the number of viable embryos by the total number of embryos analyzed. Previous studies showed that the percentage of seed viability estimated by the tetrazolium test was very similar to the percentage of seed germination in several species of orchids [77,78]. The effects of treatments on fruit set and seed viability were tested with a GLM with pollination treatment and populations as fixed factors and individuals as a random-effect factor. Fruit set and seed set were respectively log- and square-root transformed before the analyses and, when necessary, analyses were followed by ad-hoc comparisons using the Scheffe test or the *t*-test.

Two measures of postmating isolation and a combined index were calculated as follows [79]. First, we estimated postmating prezygotic isolation as the proportion of interspecific fruits formed after interspecific crosses relative to the proportion of fruits formed after intraspecific crosses:

$$\begin{array}{ll} \;{\text{Post}}_{\text{prezygotic}}=1‒ & \left(\text{fruit set formed in interspecific crosses}\right)/\\ & (\text{average fruit set formed in intraspecific}\\ & \text{parental crosses}). \end{array}$$

Likewise, postmating postzygotic isolation was calculated as the proportion of viable seeds obtained from interspecific crosses relative to the proportion of viable seeds obtained from intraspecific crosses:

$$\begin{array}{ll} \;{\text{Post}}_{\text{postzygotic}}=1‒\;( & \%\;\text{of viable seeds formed in interspecific}\\ & \text{crosses})/(\%\;\text{of viable seeds formed in}\\ & \text{intraspecific parental crosses}). \end{array}$$

The strength of postmating barriers varied between 0 (no isolation) and 1 (complete isolation). When interspecific crosses performed better than intraspecific crosses, the barrier was set to zero.

### Niche models

Niche models were generated using the Maxent algorithm [80], which estimates a target probability distribution by finding the probability distribution of maximum entropy subject to a set of constraints that represent the incomplete information about the target distribution [81]. The Maxent algorithm was chosen because it is specially recommended when a low number of presences (<30) is available [82-85]. A total of 36 localities obtained from fieldwork were considered: 19 from *E. madsenii* and 17 from *E. rhopalostele* (Additional file 5: Figure S2). No records from distribution databases were included because of the difficulty of checking the accuracy of the identifications. No niche models were constructed for *E. falcisepalum* owing to the low number of known localities. GIS layers used in the construction of niche models included the 19 climate data variables from Worldclim (http://www.worldclim.org/) with a 30 arc-second resolution (approximately 1 km^2^) and altitude obtained from SRMT (http://srtm.csi.cgiar.org/) at the same spatial resolution. Only uncorrelated environmental variables were considered. To select final variables, a jackknife was carried out in order to select variables higher than 0.75 using the AUC predicted performance.

The species’ niche was also projected on a future climate scenario for the end of the twenty-first century, according to the SRES-A1B, with a scenario reflecting an important increase in CO_2_ concentration as a result of non-restricted CO_2_ emission (Intergovernmental Panel on Climate Change, Special Report on Emission Scenarios, scenario A1, [85]). Future areas of climatic suitability were compared with present-day suitable areas and present distribution by calculating the percentage of area lost under a scenario of unlimited dispersal. Fitness of the models was assessed using AUC of a receiver-operating characteristics plot [86,87]. To obtain a binary map, the threshold of the resulting models was determined as the minimum predicted value that included all records of each species, so that a 0% omission error (proportion of observed presences incorrectly predicted) was attained [81]. The resulting maps illustrated the minimal predicted area (MPA) predicted for 100% of the presences [88]. By assigning a 0% omission error, we ensured that all known populations were included in the predicted area.

## Availability of supporting data

The data sets supporting the results of this article are included within the article and in GenBank (http://www.ncbi.nlm.nih.gov). The AFLPs matrix and reproductive tables are available from the authors upon request and will also be available on the orchid page of the UTPL website that is currently under construction (http://www.utpl.edu.ec/).

## Competing interests

The authors declare that they have no competing interests.

## Authors’ contributions

IM carried out the molecular genetic studies, field experiments and drafted the manuscript. DD constructed the ecological niche models. LR and CL participated in the field experiments. All authors read and approved the final manuscript.

## Supplementary Material

Additional file 1: Table S1 Nucleotide variation in the six cpDNA regions (*trnL-trnF, rps16*, *rpoC*, *psbk-psbI*, *matK*, and *rbcl*), frequency of haplotypes and variable positions. Click here for file

Additional file 2: Table S2 GenBank accession numbers of the DNA sequences analyzed in this study. Click here for file

Additional file 3: Table S3 **(a)** Comparative information for the six cpDNA surveyed. **(b)** Comparative information for the three studied species based on the six orgDNA surveyed. Click here for file

Additional file 4: Figure S1 Posterior probabilities (*q*) for *E. madsenii* ‘MAD’, *E. falcisepalum* ‘FAL’ and *E. rhopalostele* ‘RHO’ in HZ1 analyzed with STRUCTURE and NEWHYBRIDS. Individuals identified in the field, based on morphological characters, are delimited by dashed lines. Each vertical bar represents an individual. The proportion of color in each bar represents an individual’s assignment probability, according to different categories (pure parental species, F_1_ and F_2_ hybrids, and the respective backcrosses). Click here for file

Additional file 5: Figure S2 Geographic distribution of *Epidendrum madsenii*, *E. rhopalostele*, and *E. falcisepalum* based on reported localities plus the localities sampled during this study. The three hybrid zones studied are highlighted in green. Click here for file

Additional file 6: Table S4 Populations studied, geographic coordinates and sampling number for genetic and flow cytometry studies. Click here for file
