# Supplementary material for: Multiple hybridization events, polyploidy and low postmating isolation entangle the evolution of neotropical species of Epidendrum (Orchidaceae)
Source: BMC Evol Biol. 2014 Feb 4;14:20. doi: 10.1186/1471-2148-14-20 (PMC3927766; doi:10.1186/1471-2148-14-20)
Supplement: Additional file 3: Table S3 — (a) Comparative information for the six cpDNA surveyed. (b) Comparative information for the three studied species based on the six orgDNA surveyed. [file 1471-2148-14-20-S3.doc]

Table S3a. Comparative information for the six orgDNA surveyed. bp: base pairs; H_d_: Haplotype diversity; π: nucleotide diversity.

|  | **ndhF** | | | **Rps16** | | | **rpoC** | | |
| --- | --- | --- | --- | --- | --- | --- | --- | --- | --- |
|  | **HZ1** | **HZ2** | **HZ3** | **HZ1** | **HZ2** | **HZ3** | **HZ1** | **HZ2** | **HZ3** |
| **N. Bp** | 749 | 749 | 749 | 674 | 674 | 674 | 461 | 461 | 461 |
| **N. Invariable bp** | 728 | 728 | 730 | 644 | 648 | 645 | 459 | 459 | 457 |
| **N. Variable bp** | 21 | 21 | 19 | 15 | 13 | 16 | 2 | 2 | 4 |
| **N. Singleton bp** | 0 | 2 | 0 | 0 | 0 | 3 | 0 | 0 | 2 |
| **N. parsimony informative bp** | 21 | 19 | 19 | 15 | 13 | 13 | 2 | 2 | 2 |
| **N. haplotypes** | 7 | 4 | 3 | 4 | 2 | 4 | 2 | 2 | 4 |
| **Hd** | 0.1224 | 0.01161 | 0.01246 | 0.00985 | 0.00904 | 0.01020 | 0.00207 | 0.00199 | 0.00230 |
| **π** | 0.768 | 0.526 | 0.583 | 0.644 | 0.460 | 0.557 | 0.478 | 0.460 | 0.542 |
| **GC content** | 0.295 | 0.296 | 0.296 | 0.321 | 0.320 | 0.321 | 0.430 | 0.430 | 0.431 |
|  | **psbK-psbI** | | | **matk** | | | **rbcl** | | |
|  | **HZ1** | **HZ2** | **HZ3** | **HZ1** | **HZ2** | **HZ3** | **HZ1** | **HZ2** | **HZ3** |
| **N. Bp** | 441 | 441 | 441 | 822 | 822 | 822 | 532 | 532 | 532 |
| **N. Invariable bp** | 345 | 347 | 347 | 813 | 812 | 812 | 529 | 529 | 528 |
| **N. Variable bp** | 12 | 10 | 10 | 9 | 10 | 10 | 3 | 3 | 4 |
| **N. Singleton bp** | 0 | 0 | 0 | 2 | 3 | 3 | 0 | 0 | 1 |
| **N. parsimony informative bp** | 12 | 10 | 10 | 7 | 7 | 7 | 3 | 3 | 3 |
| **N. haplotypes** | 3 | 2 | 2 | 4 | 5 | 4 | 2 | 2 | 3 |
| **Hd** | 0.01422 | 0.1288 | 0.0141 | 0.00420 | 0.00416 | 0.00455 | 0.00270 | 0.00259 | 0.00279 |
| **π** | 0.568 | 0.460 | 0.500 | 0.542 | 0.515 | 0.557 | 0.478 | 0.460 | 0.511 |
| **GC content** | 0.262 | 0.260 | 0.260 | 0.312 | 0.312 | 0.312 | 0.417 | 0.416 | 0.417 |

Table S3b. Comparative information for the three studied species based on the six orgDNA surveyed. H: number of haplotypes (N_var_: number of variable sites; N_par_: number of parsimony informative sites; H_d_: Haplotype diversity (for each region); π: nucleotide diversity.

|  | **H** | **N_var_** | N_par_ | H_d_ | π | **GC** |
| --- | --- | --- | --- | --- | --- | --- |
| **E. mad** | 9 | 12 | 3 | 0.800 | 0.00041 | 0.337 |
| ***E. rhopa*** | 1 | - | - | - | - | 0.335 |
| ***E. fal*** | 5 | 6 | 5 | 0.756 | 0.00071 | 0.336 |
| **hybrids** | 2 | 52 | 2 | 0.300 | 0.00431 | 0.336 |
