# Supplementary figures and images for: Multiple hybridization events, polyploidy and low postmating isolation entangle the evolution of neotropical species of Epidendrum (Orchidaceae)
Source: BMC Evol Biol. 2014 Feb 4;14:20. doi: 10.1186/1471-2148-14-20 (PMC3927766; doi:10.1186/1471-2148-14-20)

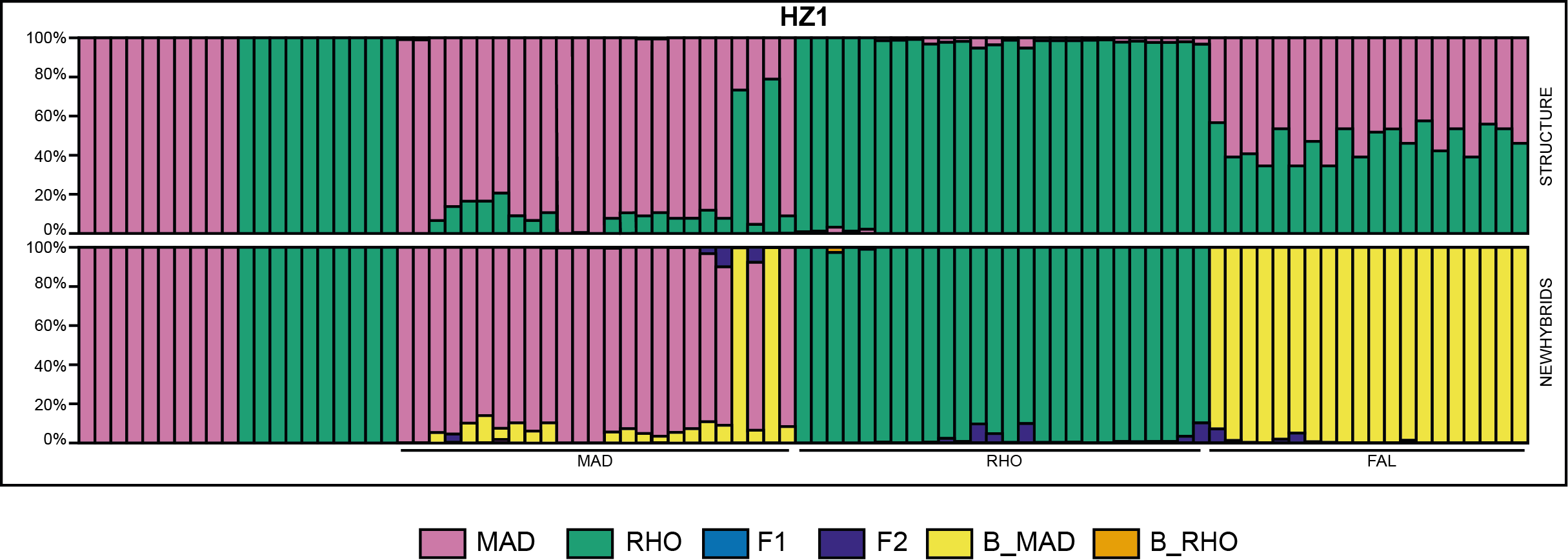

Supplement: Additional file 4: Figure S1 — Posterior probabilities (q) for E. madsenii ‘MAD’, E. falcisepalum ‘FAL’ and E. rhopalostele ‘RHO’ in HZ1 analyzed with STRUCTURE and NEWHYBRIDS. Individuals identified in the field, based on morphological characters, are delimited by dashed lines. Each vertical bar represents an individual. The proportion of color in each bar represents an individual’s assignment probability, according to different categories (pure parental species, F1 and F2 hybrids, and the respective backcrosses). [file 1471-2148-14-20-S4.tiff]

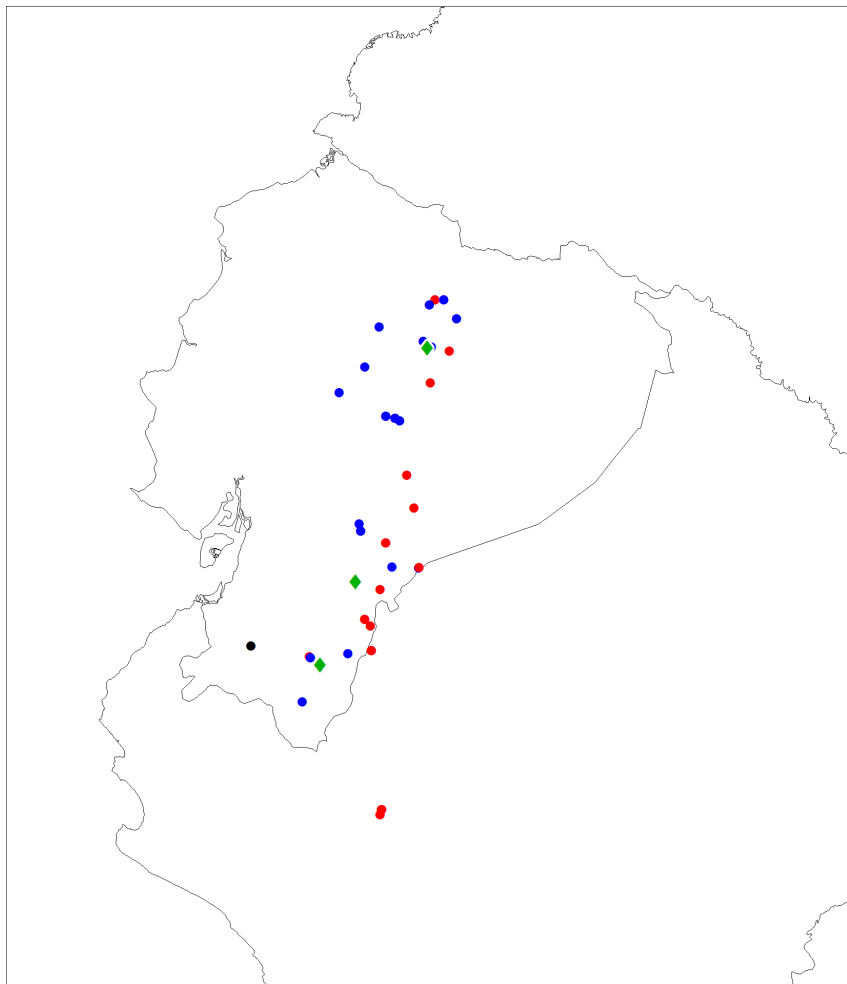

Supplement: Additional file 5: Figure S2 — Geographic distribution of Epidendrum madsenii, E. rhopalostele, and E. falcisepalum based on reported localities plus the localities sampled during this study. The three hybrid zones studied are highlighted in green. [file 1471-2148-14-20-S5.pdf]
